# Investigation of the effects of concentration and voltage on the physicochemical properties of Nylon 6 nanofiber membrane

**DOI:** 10.1038/s41598-025-88356-y

**Published:** 2025-03-29

**Authors:** Omolola E. Fayemi, Petu B. Letsholo, Hassan O. Shoyiga, Chinedu C. Ahia, Edson L. Meyer

**Affiliations:** 1 Material Science Innovation and Modelling (MaSIM) Research Focus Area, Faculty of Natural and Agricultural Sciences, North-West University (Mafikeng Campus), Private Bag X2046, Mmabatho, 2735 South Africa; 2 Department of Chemistry, Faculty of Natural and Agricultural Sciences, North-West University, Mafikeng Campus), Private Bag X2046, Mmabatho, 2735 South Africa; 3 Institute of Technology, University of Fort Hare, Private Bag X1314, Alice, 5700 South Africa

**Keywords:** Nylon 6 nanofiber, Concentration, Voltage, Spectroscopy, DFT, Chemistry, Materials science, Nanoscience and technology

## Abstract

The nanofibers of Nylon 6 at different concentrations 14 wt%, 16% wt%, and 18 wt% were fabricated using electrospinning method. The effect of concentration and voltage on the morphology of the nanofibers were assessed through the utilisation of FTIR, XRD, Raman spectroscopy, atomic force microscopy AFM, scanning electron microscopy SEM, and EDS techniques. A study on the Nylon 6 molecule using DFT (Density Functional Theory) was conducted. Nylon 6 optimized molecular structure, featuring an amine group and nitrogen atom for electrophilic reactions, exhibits high kinetic stability with a measured band gap energy of 6.60 eV and electronic chemical potential of -3.83. The diameter of the fibres increased from 76.91 to 81.98 nm when the concentration and voltage were further increased, with the respective weight percentages of 14 wt% and 18 wt%. This work reveals the influence of concentration and voltage on the physical and chemical characteristics of nylon-6 NFM and its quantum electronic properties.

## Introduction

Nylon 6 nanofiber membranes have attracted significant research interest and have a wide range of applications because of their extraordinary properties, which include enormous mechanical strength, increased porosity and high surface area. These fibres are typically manufactured using conventional techniques such as melt spinning, dry spinning, and wet spinning^1^. Due to its high level of manufacturability and wide variety of applications, this technology has demonstrated considerable potential in various sectors^2^.

Polystyrene, polyvinyl alcohol, polyvinylidene fluoride, and polyamide 6, commonly referred to as Nylon 6 are extensively utilised thermoplastic polymers in nanofiber synthesis^3^. In the energy sector, nylon 6 nanofiber membranes can be utilized as a safety device known as battery separators to prevent electrical short circuits and can serve as effective proton exchange membranes in fuel cells. They can be used for both air and water filtration to isolate contaminants such as viruses, bacteria, pollutants and particulate matter. Most importantly, nylon 6 nanofiber membranes also find applications in the biomedical field and can be used as a platform for automated drug delivery procedures, cell development, tissue engineering and repair. The properties of nylon-6 fibres, both physical and mechanical, are influenced by various parameters, which include manufacturing processes, molecular mass, and molecular weight dispersion^4^. Other relevant applications of nylon 6 nanofiber membranes are in food packaging, sensors, production of high-performance textiles such as breathable fabrics, photonics, nanocomposites, catalysts, and adsorption chemistry^5^.

Despite having great potential applications, there are still some challenges associated with nylon 6 nanofiber membrane utilization, performance and production. These challenges include the fact that they are susceptible to chemical and mechanical degradation, toxic, non-biodegradable, difficult to recycle, scalability and high production cost. Furthermore, they are prone to fouling and clogging hence, reducing their effectiveness.

To mitigate the abovementioned shortcomings and optimize the effectiveness of nylon 6 nanofiber membranes, certain criteria are crucial. One of the techniques that can significantly enhance the performance, longevity and functionality of this class of nanofiber membranes is the optimization of its developmental technique through electrospinning process. The membranes obtained from electrospun polymer fibres have a high surface area per unit mass and a very small pore size due to the small size of the fibres^6^. The electrospun fibres possess several attributes that render them suitable for a wide range of prospective applications, including optoelectronic materials, sensor, and nanocomposite materials, drug delivery systems, tissue scaffolds, protective garments, filtration and wound dressings^7,8^.

Formals initially invented the method of electrospinning in 1934^9^. Details of the procedure have been reported in other literature^10–16^. Precise control and optimization of electrospinning parameters (such as surface voltage, flow rate, the distance between the spinneret and the collector) during synthesis can be beneficial towards the actualization of good surface morphology and fibre diameter which ultimately gives rise to enhanced filtration efficiency due to improved surface area, porosity and permeability. The polymeric concentration and the voltage applied to the solution are vital parameters during electrospinning process which can be controlled and optimized appropriately, thereby making the nylon 6 nanofiber membranes to be suitable for various applications. Furthermore, the physiochemical properties of nylon 6 nanofiber membranes can be modified for specific applications by carefully tailoring the polymeric concentration and applied voltage. Optimization of these two parameters can lead to synergistic effects, resulting in improved membrane properties.

The present study investigates the development of a nylon-6 nanofiber membrane (NFM) utilising a precursor solution which consists of a mixture of formic and acetic acid. The selection of Nylon-6 was based on its favourable electrospinning properties and its solubility in both polar and nonpolar solvents. The physicochemical features of the synthesised Nylon 6 nanofiber membranes were analysed using XRD (x-ray diffraction), Transformation Infrared (FTIR) spectroscopy, Scanning Electron Microscopy (SEM), and Energy Dispersive Spectroscopy (EDS)^6^. Furthermore, the vibrational modes and physical features such as the surface roughness and topography were examined using confocal Raman spectroscopy and Atomic Force Microscopy (AFM). The present study also examined the impact of solution molarity, applied potential, and needle tip-to-collector distance on both the structure and average dimensions of the electrospun NFM mats. In addition, it is worth noting that previous studies have not adequately explored the theoretical aspects of the electronic characteristics of NFM. Therefore, in addition to our investigation on Nylon 6 NFM, we employed density functional theory (DFT) to mathematically establish the optimised molecular geometries and electronic characteristics.

## Methodology

### Materials and equipment

The materials used for this study includes Nylon 6 pellets (99.85%, Sigma Aldrich, South Africa), acetic acid ($\:{\text{C}\text{H}}_{3}\text{C}\text{O}\text{O}\text{H})$ and formic acid $\:\left(\text{H}\text{C}\text{O}\text{O}\text{H}\right)$ (98–100%, Sigma Aldrich, South Africa). All reagents were of analytical grade and were used without further purification. The equipment utilised are hot plate with stirrer, stirrer bar, and electrospinning machine, while the techniques employed for analysis include FTIR, XRD, SEM-EDS, Raman, and AFM.

### Synthesis method

#### Preparation of Nylon 6 solution

The concentration of the nylon 6 polymer solution is critical, as it directly influences viscosity and surface tension, which are essential for fibre formation. Increased concentrations generally elevate viscosity, thereby improving the solution’s capacity to produce continuous fibres instead of droplets. Prior research indicates that inadequate viscosity at low concentrations (e.g., 10 wt% and below) results in suboptimal fibre formation and heightened bead creation. In contrast, concentrations exceeding 10 wt% have been shown to produce smoother fibres, attributed to enhanced viscoelastic properties that mitigate surface tension^17,18^. A study by Tariq et al.^18^ indicated that optimising concentration to approximately 23 wt% in polyvinylidene fluoride (PVDF) solutions led to enhanced fibre morphology and decreased bead formation. Thus, selecting the appropriate concentration to attain the desired fibre characteristics is essential. Our polymer solution preparation was developed following the aforementioned principle.

A solution consisting of formic and acetic acid in a 1:1 ratio was prepared in a 10 ml beaker. The Nylon 6 pellets were subsequently dissolved in the prepared solvent, at varied concentrations: 14, 16, and 18 wt%. Subsequently, the mixture was subjected to overnight agitation using a stirring apparatus to achieve complete homogenization^7^.

#### Preparation of Nylon 6 nanofiber membrane (electrospinning method)

**Fig. 1:**
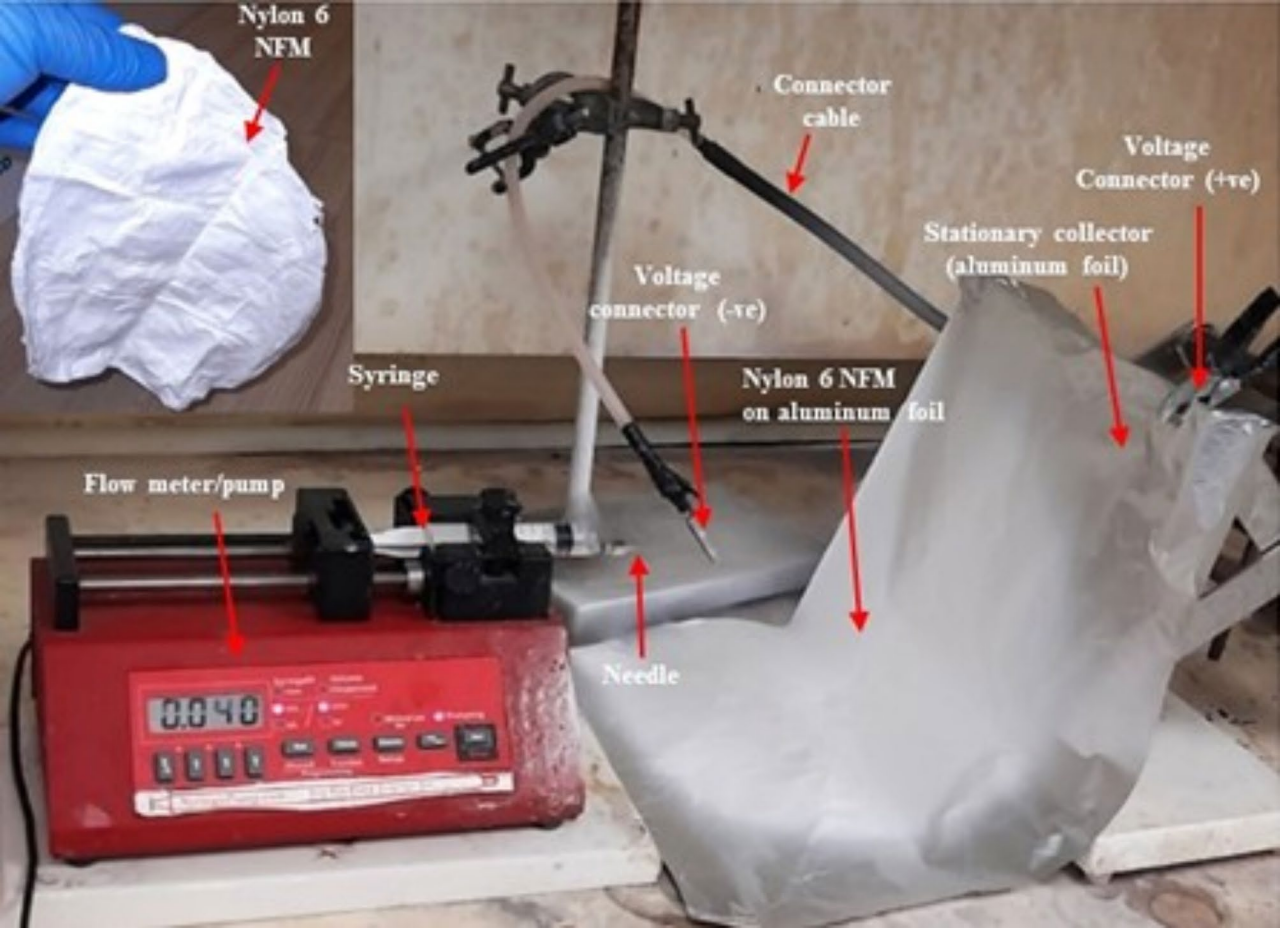
Electrospinning setup and synthesised NFM (inset).

Figure 1 illustrates the experimental arrangement employed for the electrospinning process of nylon 6 nanofiber membrane. In summary, a 5 ml syringe was filled with a solution of nylon 6 and fitted with a capillary tip that had an inner diameter of 0.7 mm. The nozzle tip was positioned 15 cm from the collector, and a voltage of 20 kV was applied, resulting in a flow rate of 0.04 ml/h. In addition, the nozzle tip was positioned 12 cm away from the collector, and a potential of 25 kV was applied, with a flow rate of 0.02 ml/h. The collector was covered with an aluminium foil, facilitating the deposition and growth of a nanofiber membrane^7^.

It must be noted that the distance between the needle tip and collector was carefully selected before electrospinning process commenced, as it affects the time available for solvent evaporation before the fibers reach the collector. An optimal distance allows for sufficient drying of the polymer jet, leading to solid fibres upon collection. Zahra et al.^17^ demonstrate this in their work that increasing the needle tip-to-collector (i.e., spraying) distance can lead to thinner fibres as it allows more time for solvent evaporation, reducing fibre diameter^17,19^. However, excessively long distances may result in increased fibre diameter due to additional stretching and potential instability of the jet. For instance, studies utilizing a needle-to-collector distance of approximately 15–20 cm have shown favourable results in producing uniform nanofibers with minimal defects^20^.

Similarly, the applied voltage in electrospinning is an important factor, as it generates the electric field needed to attract the polymer solution from the needle tip to the collector. An increase in voltage amplifies the electrostatic forces exerted on the solution, potentially leading to enhanced jet elongation and thinning. Studies show that raising the voltage typically results in a reduction of fibre diameter, attributed to the enhanced stretching of the polymer jet. A study indicated that at voltages of 20 kV and higher, the production of smoother and thinner fibres was consistently observed, whereas lower voltages led to bead formation due to inadequate force to overcome surface tension^18^. This is consistent with results from additional studies indicating that optimal voltage settings are essential for attaining uniform fibre morphology free from defects^20^.

### Characterisation

The morphology of the nylon 6 nanofiber membrane produced through electrospinning was analysed using a scanning electron microscope (SEM) with a Quanta FEG 250 microscope. Also, a WiTec alpha 300RA + microscope was used to obtain 2D and 3D AFM micrographs that display the surface characteristics of the Nylon 6 nanofiber. Image J software was employed to determine the dimension of the nanofiber’s diameter using the SEM images. The investigation of crystallinity and changes in crystal structure resulting from the conversion into nanofiber was conducted using powder XRD technique. The diffractograms were acquired using a Rontgen PW3040/60 X’Pert Pro diffractometer, with a scan rate of 2 min^− 1^ and a diffraction angle range of 10^◦^ − 90^◦^. Fourier transform infrared (FTIR) spectra of the nanofiber membrane were obtained using a Bruker Alpha (Opus Alpha-P) spectrophotometer equipped with a universal ATR (attenuated total reflectance) accessory. The spectra were obtained in the wavenumber region from 4000 to 400 cm ^− 1^. Furthermore, a confocal Raman spectroscopy investigation was conducted on the samples using a WiTec alpha 300RA_+_ confocal Raman microscope and a laser wavelength of 532 nm as excitation source.

### Computational methods

All computations were performed using the Gaussian 09 software. The compound’s optimised geometrical characteristics and frequencies of vibration were calculated using the DFT/RB3LYP^22^ method with the 6-311 + + G (d, p) basis set. The scale factor 0.9613^23^ was employed to ensure a balance between empirical and theoretical harmonic frequencies. The findings were examined using the Gauss view 6.0 software for molecular visualisation^23^. A DFT technique utilising an identical basis set has been proposed for the computation of molecular electrostatic surface potential (MESP), as well as electronic characteristics like HOMO-LUMO interactions and reactivity indices.

## Results and discussion

The Nylon-6 nanofiber is categorised as a copolymerized form of nylon. It has a low melting point and has outstanding mechanical characteristics, such as resistance to wear, oil, and heat. Therefore, it is ideal for constructing single- and multiple-strand fibres, films, and nets^24^. This study describes the production of a Nylon-6 nanofiber membrane through a two-step synthesis process. Firstly, the initial step involves preparing a mixture of solvents and dissolving the nylon 6 pellets. Additionally, the process involves electrospinning and depositing a nylon 6 solution onto an aluminium collector. This work explores how the synthesis parameters impact the physicochemical characteristics of the electrospun nanofiber membrane, specifically focusing on its morphology and cross-sectional area. Several characterization techniques were employed to validate the synthesis and fabrication of nanofiber. Also, the theoretical investigation of its electronic properties like HOMO-LUMO interaction, energy gap, MESP, and other quantum chemical parameters using the DFT technique. The resulting NFM is a thin, white, pliable substance with a smooth, velvety feel reminiscent of a serviette paper. Figure 1 displays the pictorial representation of the synthesis method used for producing the electrospun nylon 6 nanofiber membrane, together with the resulting NFM (shown in-inset).

### Fourier transform infrared (FTIR) spectroscopy

**Fig. 2:**
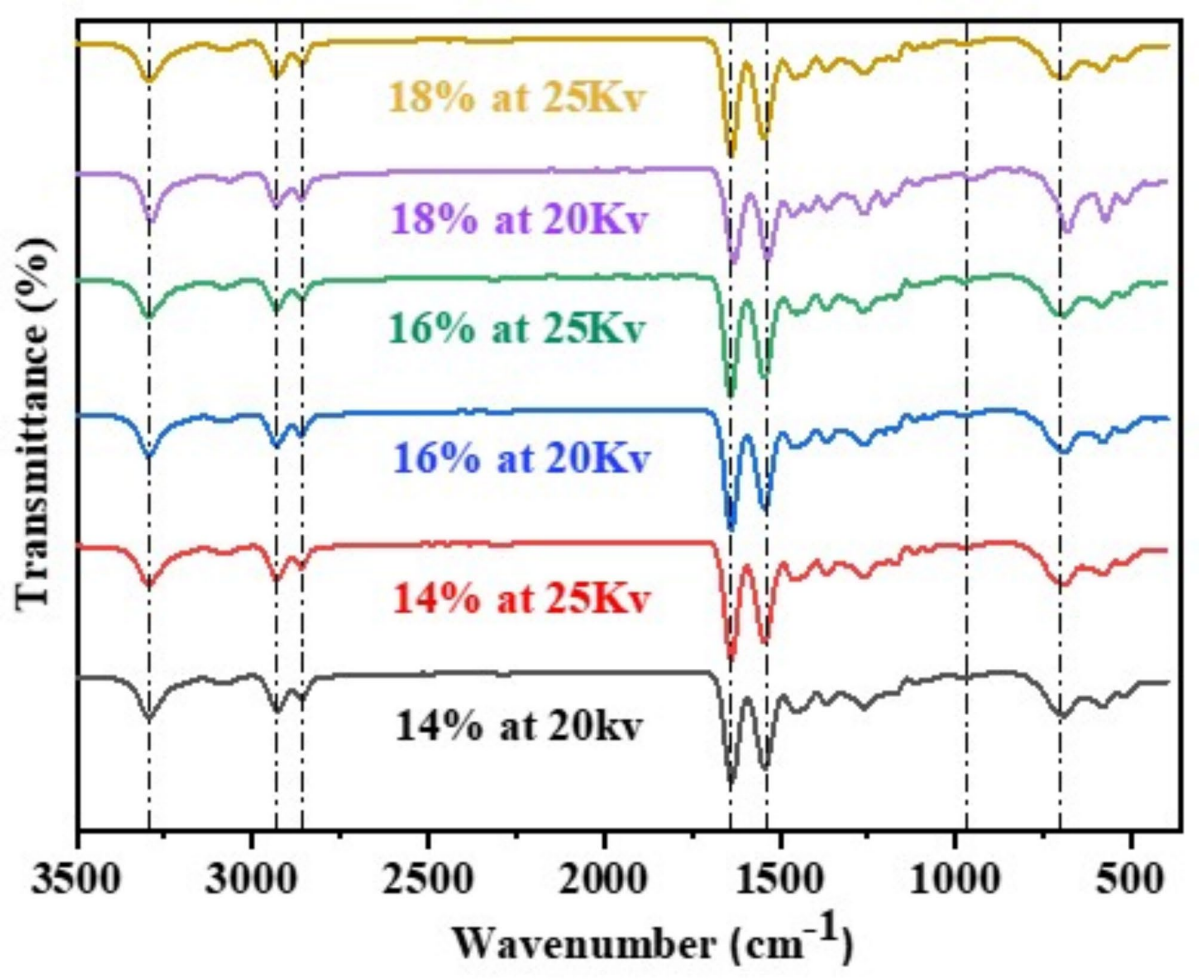
FTIR spectra of Nylon 6 NFM electrospinned at different concentrations and applied voltage.

Fourier Transform Infrared Spectroscopy (FTIR) is employed to identify the infra-red peaks corresponding to the frequencies of vibration of atoms present in electrospun nylon-6 NFM. The FTIR investigation has identified absorption peaks that match to the frequencies of vibration in the electrospun NFM. The spectra of the electrospun nylon 6 nanofiber membranes exhibited a nearly high degree of similarity, indicating the presence of distinct absorption bands associated with amide groups and methylene parts of polyamide. Figure 2 displays the ATR-FTIR spectra of the electrospun nylon 6 nanofibers, spanning from 3500 cm⁻¹ to 450 cm⁻¹ in frequency range^15^. The spectrum of the nylon 6 nanofibers exhibited prominent peaks at distinct wavenumbers, such as the N-H (3293 cm⁻¹) stretching, -CH₂ (2932 cm⁻¹) stretching, -CH- (2859 cm⁻¹) symmetric stretching, -C = O (1640 cm⁻¹) stretching, and N-H (1539 cm⁻¹) bending vibration^15^. In addition, there were notable peaks such as Amide IV and C-CO stretch seen at 973 cm⁻¹, (CH₂ > 4, wag) at 698 cm⁻¹, and Amide II, N-H out-of-plane bend acquired at 588 cm⁻¹, which correspond to the stretching of the C-N bond, bending of the CO-N-H bond, and bending of the N-H bond out of the plane, respectively. These peaks were likewise detected in all the spectra of additional nylon 6 versions produced at varying concentrations and voltages. No substantial change in the wavelength was detected for any of the synthesised nylon 6 NFMs. Suggesting their inherent molecular stability and great resistance to alterations to specific external forces. This observation may be attributed to the robust intermolecular contact between the nitrogen and oxygenic functionalities, which results in the generation of intermolecular H-bonding. Thus, the identical IR spectra of the electrospun nylon 6 NFMs suggested that the molecular frameworks of the nylon-6 NFMs remained unchanged despite changing concentration and applied voltage during electrospinning^16^.

### Xray diffraction analysis

**Fig. 3:**
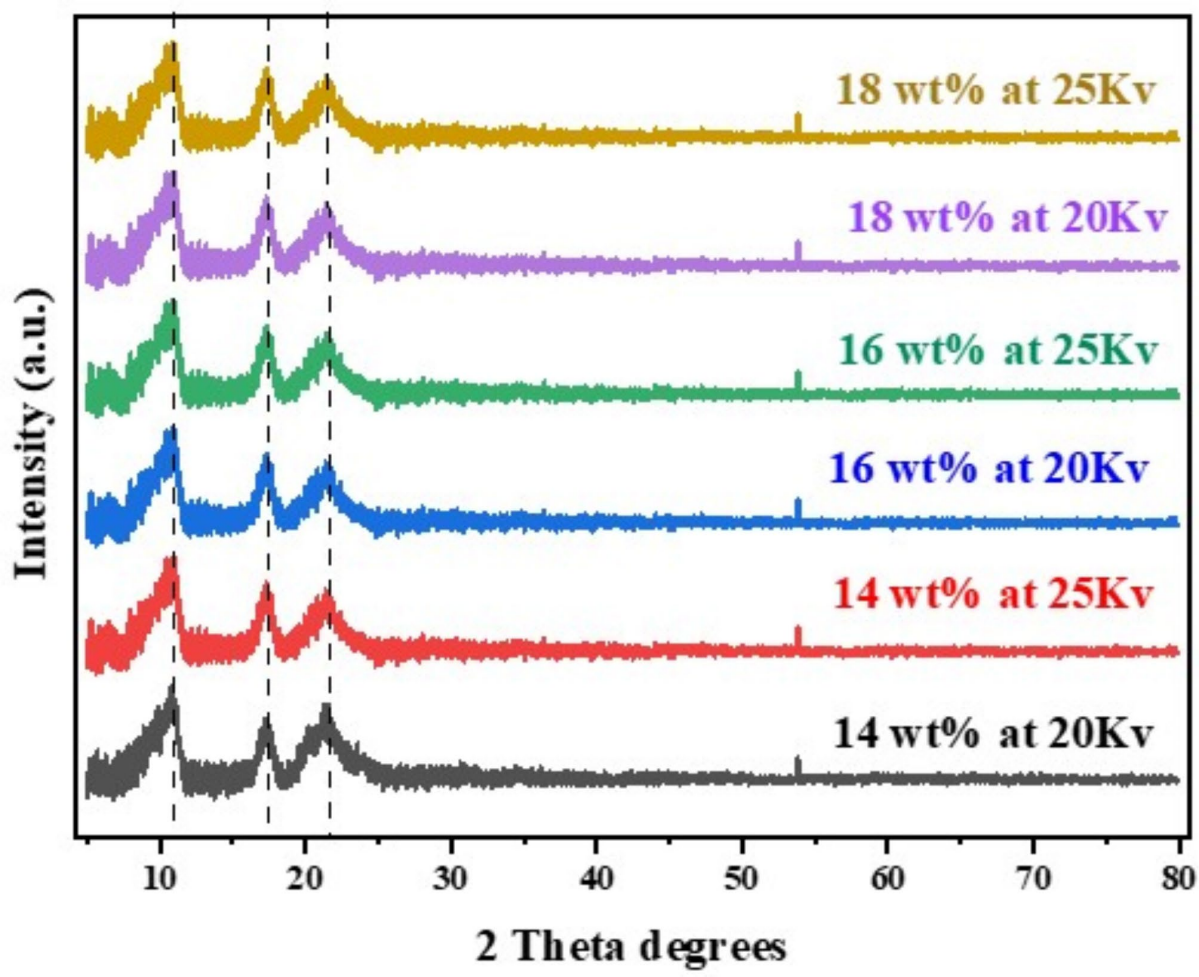
XRD spectrum of synthesized nylon 6 nanofibers at varying concentration and voltage.

We performed a study on the crystalline characteristics and alterations in crystallinity that arise during the synthesis of nylon-6 NFM. The diffraction pattern for electrospun nylon 6 NFM was measured in the angular range of 5° to 80°. The XRD pattern in Fig. 3 illustrates the results obtained from nanofiber weight percentages of 14 wt%, 16 wt%, and 18 wt%. The diffraction peaks of the nanofibers were detected at 2θ values corresponding to Bragg’s angle in the regions of 11.2°, 17.23°, and 21.5°. The peaks seen at 2θ values ranging from 20° to 25° are characteristic of nylon-6 NF and have been extensively established in the literature^25^. The α1 peak (2θ = 21.5°) is caused by the separation between chains that are linked together by hydrogen^15^.

The presence of these peaks indicates that the molecular diffractogram pattern of nylon 6 has characteristics of semi-crystallinity. Regarding literature, the XRD diffractogram of pure nylon-6 exhibits crystalline characteristics. During this experiment, the XRD diffractogram of the nylon-6 nanofiber exhibited a partially crystalline structure. The findings indicate that nylon-6 undergoes a transition into a semi-crystalline state, rather than maintaining a purely crystalline structure. The phenomenon of electrospinning may have played a role in causing this modification in its structure. The primary reason for this observation might be attributed to the amorphization that takes place during the electrospinning process^25^. This phenomenon is believed to occur during the synthesis of nylon-6 NFM. The precursor solution solidifies when it is stretched and pulled towards the collector. The solvent molecules undergo evaporation during the transformation process, while the intermolecular bonds between the polymer molecules are preserved and subsequently reinforced. The phenomenon of ionic mobility, accelerated by an electric field, prevents the nylon-6 molecules from arranging themselves in a manner conducive to forming a crystal structure. This rapid movement may be the reason behind the semi-crystalline nature of the Nylon 6 nanofiber polymer^14^. The diffractogram of all nylon 6 nanofibers exhibits a high degree of similarity, as seen in Fig. 3; Table 1.

The crystallinity of Nylon 6 is affected by structural parameters, including the percentage of crystallinity and microstrain, which dictate the deformation behaviour of the polymer. Microstrain denotes lattice distortions within crystalline regions, typically resulting from uneven stress distribution during fibre formation and solvent evaporation in the electrospinning process. An increased microstrain value signifies enhanced lattice distortion, which minimises crystallinity and influences the material’s physical properties. Electrospun nylon 6 exhibits broader diffraction peaks, indicating elevated microstrain levels relative to solution-cast films^26^. In the synthesised nylon 6 nanofibre, a lower lattice distortion is observed as a result of the low microstrain value (Table 1). Thus, corroborating the partial crystalline properties observed in the synthesised nylon 6. The percentage of crystallinity quantifies the proportion of polymer chains that establish ordered crystalline regions, where increased crystallinity correlates with enhanced mechanical strength and thermal stability. The parameters of electrospinning can influence chain alignment during jet stretching, thereby impacting the extent of crystallisation. Electrospun nylon 6 nanofibers exhibit lower crystallinity than solution-cast films, with as-electrospun nylon 6 displaying a crystallinity ranging from approximately 22–27%, contingent upon the confinement conditions during the electrospinning process^14^. On the contrary, the electrospun nylon 6 in the present work shows a higher percentage of crystallinity ranging between 77.43 and 87.24%. This suggests that the crystalline arrangement of nylon 6 remains intact across all concentrations and voltage levels^15^.

**Table 1:** Crystallite size and d-spacing of nanofibers at different concentrations and applied voltage.

Concentration (wt%)	Voltage @ 20 kV				Voltage @ 25 kV			
	Diameter (nm)	d-spacing (nm)	Microstrain	Crystallinity (%)	Diameter (nm)	d-spacing (nm)	Microstrain	Crystallinity (%)
14	4.23	0.85	98.50	81.25	4.23	0.85	94.90	83.93
16	4.02	0.86	99.99	77.43	3.91	0.85	96.70	87.24
18	4.18	0.86	96.09	83.01	4.05	0.86	99.48	77.72

### SEM analysis and EDS

**Fig. 4:**
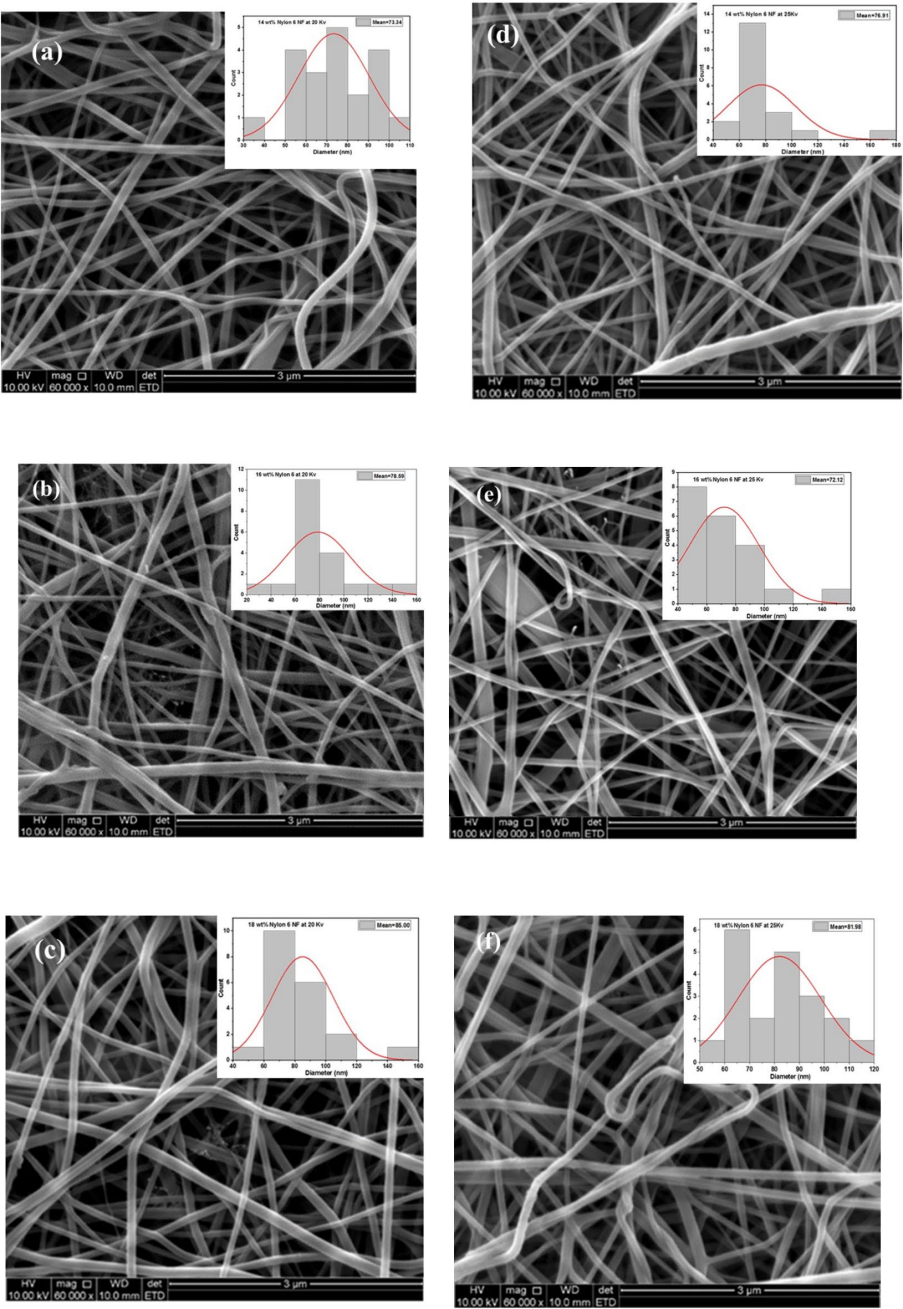
SEM images of electrospun nylon 6 nanofibers at different concentration and voltages (**a**) 14 wt% @ 20 kV, (**b**) 16 wt% % @ 20 kV, (**c**) 18 wt% @ 20 kV, (**d**) 14 wt% @ 25 kV, (**e**) 16 wt% % @ 25 kV, and (**f**) 18 wt% @ 25 kV with their respective fibre diameter size distribution (nm) (inset).

SEM micrographs and the average fibre diameter distribution for nylon 6 nanofibers electrospun at concentrations of 14, 16 and 18 wt% are displayed in Fig. 4 (a-f). The electrospinning process was carried out using applied potentials of 20 and 25 kV, with a needle end-to-screen collector spacing of 15 cm. Also, for ease of comparison, the SEM micrographs were obtained at the same magnifications. The obtained micrographs showed no beads formed on the nanofibers or during electrospinning. Smooth nanofibers were uniformly distributed with thin length and cylindrical with an average diameter of 73.34 nm at 14 wt% concentration and 20 kV^7^. As the concentration increased from 16 to 18 wt%, the fibres were slightly thicker, with average diameters of 78.59 nm and 85 nm, respectively at 20 kV. The structural appearance of the electrospun nylon 6 nanofibers changed as the fibre diameter rose progressively with increasing concentration. This phenomenon arises due to the increased presence of polymer chains in the solution, resulting from higher polymer concentrations. Consequently, this facilitates the generation of thicker fibres^6^. It must, however, be noted that the concentration of the polymer solution is of vital importance in the electrospinning process. This parameter has a significant impact on the physical and morphological characteristics of nanofibers made from various polymers^27^. Electrospun nanofibers typically exhibit three morphological variants: bead-on-string, smooth fibres and bead. Most applications prefer nanofibers that are extremely thin, elongated, and have a smooth surface^3^. Figure 4 (d-f) shows an interesting structure at 16 wt% (a) and 18 wt% (b), which is an extremely thin, spider-web-like network that is dispersed irregularly among the usual nanofibers^3^. The development of the spider-web morphology may be ascribed to the hydrogen bonding that occurs between the amide groups (CO-NH) of the main polyamide chain and the oligomeric or monomeric ionic species (CONH₂⁺). The production of oligomeric and monomeric ions in the solution is attributed to the interaction between formic acid and lactam, which leads to the formation of a continuous succession of short oligomers and monomers. As can be observed, one of the major elements influencing the production of spider-net structures is polymer concentration. Higher nanofiber concentrations of 16 wt% and 18 wt% revealed the formation of spider-net^16^.

The existence of these hydrogen bonds enhances the structural integrity of the fibres and affects their mechanical properties. The spider-web-like morphology possesses a significant interfacial area-to-volume ratio, thereby augmenting their utility in filtration and water harvesting through enhanced surface interactions with droplets, as well as in tissue engineering. Enhanced fibre connectivity improves filtration efficiency and mechanical durability in membrane filtration. The web-like structure emulates the extracellular matrix, facilitating cell attachment and proliferation, as utilised in tissue engineering. In sensors, an augmented surface area enhances sensitivity and responsiveness in chemical or biosensors^28,29^. The aforementioned outcomes are derivatives of the unique structure of electrospun fibres, which are frequently engineered to replicate natural structures like spider webs^30^.

Moreover, based on the principles of thermodynamics and kinetics. The long-term stability of hydrogen bonds affects the energy landscape of fibre development, influencing the alignment and interaction of polymer chains throughout the electrospinning process. A high density of polymer chains at elevated concentrations promotes stronger intermolecular contacts, resulting in improved structural integrity and distinctive morphologies^2^. Moreso, solvent choice profoundly influences morphology by dictating the degree of solvation and chain entanglement. As an illustration, a moderately polar solvent (e.g., formic acid) can offer adequate solvation to facilitate consistent stretching. Nonetheless, partial desolvation during jet stretching results in irregular fibre distribution and the formation of a “spider web-like” pattern^31^. This spider-net-like feature has a high interfacial area-volume ratio, which expands the applications of nanofibers^14^.

Electrospinning occurs when the voltage supplied exceeds the interfacial energy of a droplet of polymeric solution. Higher voltage, in general, leads to more considerable stretching of the jet due to an increase in Columbic force produced by the charges. Increasing the applied voltage can lower fibre diameters owing to greater jet stretching^32^.

Fluid dynamics is an additional component that must be examined to comprehend the jet behaviour of the polymer solution during electrospinning. This is regulated by factors like surface tension, viscosity, and electric field intensity. Surface tension influences the stability of the Taylor cone from which the jet emanates. Consequently, increased surface tension can stabilise larger droplets, resulting in thicker fibres. Solutions with elevated viscosity generally yield smoother fibres devoid of beads, owing to less jet instability, and conversely^2^. Furthermore, flow rate generally impacts the volume of polymer solution dispensed to the tip, hence affecting fibre diameter and morphology. As the solution is pushed into fibres, shear forces exert influence on the alignment and orientation of the polymer chains. The electric field intensity is another parameter worth considering. As voltage increases, it enhances jet stretching but may also induce whipping instability if inadequately regulated, resulting in differences in fibre morphology. The interaction between electrical forces and fluid mechanics is essential for optimising contexts to attain desired morphologies. These dynamics underscore that attaining the desired features of nanofibers necessitates meticulous regulation of various associated parameters^2,13,33^.

The SEM images in Fig. 4 (d-f) show 14, 16 and 18 wt% electrospun nylon 6 nanofiber with a voltage supplied at 25 kV and a nozzle-tip distance of 12 cm. As shown in Fig. 4 (d), when the applied voltage was 25 kV at 14 wt%, the fibres were smooth and more branched with no beaded formation and their diameter showed a slight increase as compared to when it was electrospun at 20 kV (Fig. 4a). When the concentration increased to 16 wt%, slightly beaded fibre with a spider-net morphology was formed and fibre diameter decreased slightly to 72.12 nm (Fig. 4e) as compared to a similar concentration at 20 kV. The appearance of beaded fibres has been extensively reported^34^. They were considered as “by-products” frequently produced during the process of electrospinning. The occurrence of beaded fibres has been linked to the fragility of the polymer solution jet, the viscosity of the solution, the total charge the volume carried by the electrospun jet, and the surface tension of the solution. The greater viscosity aided the manufacturing of beadless fibres^35,36^. This was once again demonstrated by the process of electrospinning nylon-6 nanofibers. When the concentration was increased to 18 wt%, fibre diameters increased to 81.98 nm (Fig. 4f) which decreased as compared to 18 wt% at 20 kV. This variation may be ascribed to the fact that increased concentration results in smaller nanofiber diameters despite higher voltages^35^. By adjusting the molarity of the polymer solution, it is possible to control the size of the electrospun nylon-6 nanofiber membrane (NFM). However, the variation of fibre sizes is quite vast. Other researchers have also discovered a wide range of sizes in electrospun polymer fibres, as reported by various publications^37^. From our perspective, the broad size distribution can be attributed to a minimum of three factors: (1) The electrospinning process, driven by a high-voltage power supply, may cause uneven disintegration of polymer droplets, leading to a wide distribution of fibre sizes. (2) The formation of many subsidiary drips after the breakdown of the solution jet may contribute to the broad distribution of fibre sizes^1^. (3) Various factors, including temperature, surface tension, relative humidity potential, solution molarity, and solvent, can affect the formation of fibres. The inherent variability in these factors recorded in Table 2 seen during the studies (perhaps unavoidable) could result in an increased spread of fibre sizes.

The elemental analysis of the nylon 6 nanofiber also shows that the synthesised materials all contain carbon, nitrogen, and oxygen in approximately the same amount with a slight difference. This difference could be a result of interference of elemental impurities obtained during the electrospinning process. The elemental composition is represented in Table 3.

**Table 2:** Size distribution of nanofibers at different concentrations and applied voltages.

Concentration (wt%)	Voltage @ 20 kV	Voltage @ 25 kV
	Diameter (nm)	Diameter (nm)
14	73.34	72.12
16	78.59	76.91
18	85.00	81.98

**Table 3:** EDS elemental composition of the synthesised nanofibers.

		Elemental composition (%)					
Sample (wt%)	Voltage (kV)	C	*N*	O	Si	Ca	Total
14	20	73.25	11.73	14.84	0.08	0.1	100
16	20	71.93	12.1	15.88	-	0.09	100
18	20	71.45	13.33	15.22	-	-	100
14	25	73.08	11.91	15.01	-	-	100
16	25	71.95	13.04	15	-	-	100
18	25	69.29	13.99	16.72	-	-	100

### AFM microscopy

**Fig. 5:**
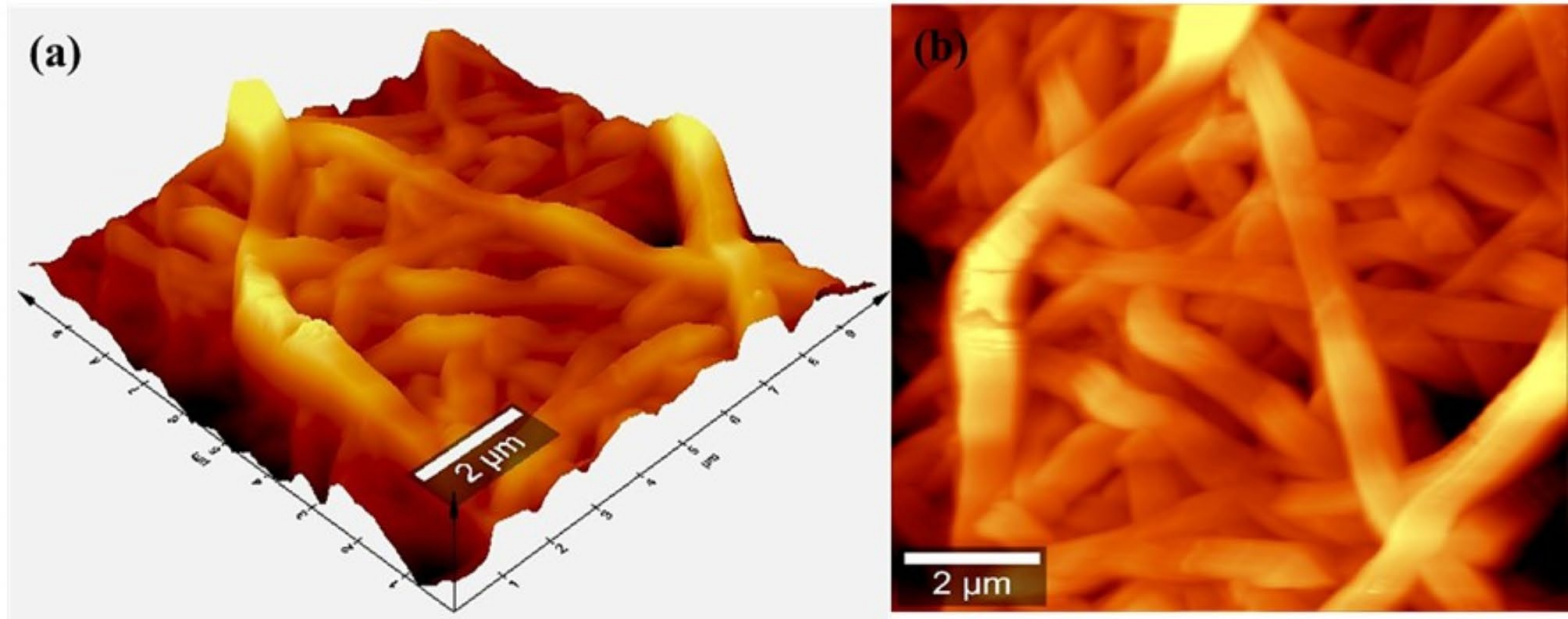
Illustrates the (**a**) 3D and (**b**) 2D AFM micrographs showing the surface morphology of Nylon 6 NFM.

Figure 5 (a) and (b) are the 3D and 2D AFM images of the nylon 6 NFM acquired across a scan area of 10 μm × 10 μm on the sample using tapping mode scanning condition. The micrograph was obtained from a WiTec alpha 300RA_+_ microscope. A moderate scan rate of 0.2 Hz was enabled for an image resolution of 256 pixel to minimize stiffness caused by damping of the cantilever. The micrograph extracted from AFM reveals a nonuniform corrugated surface morphology with scaffold-like features. The nanofibers were clearly visible in the image with mean diameter measuring ~ 95 nm as determined from a line scan. The diameter value obtained from AFM measurements for the nanofiber corresponds with measurements obtained from SEM. A root means square roughness (R_rms_) of 1.43 ± 0.06 μm was obtained from the sample by measuring a scan area of 100 μm^2^. The increased value of surface roughness measured for the nanofiber can be beneficial by acting as a favourable platform for cell nucleation due to improved adhesion, while aiding enhanced crystal growth, wettability, and hence the contact angle of a liquid. Further analysis of the image obtained from the nanofiber using AFM yielded a skewness value of -0.2456, indicating that the height distribution of the nanofiber is contorted above the mean plane.

### Raman spectroscopy

**Fig. 6:**
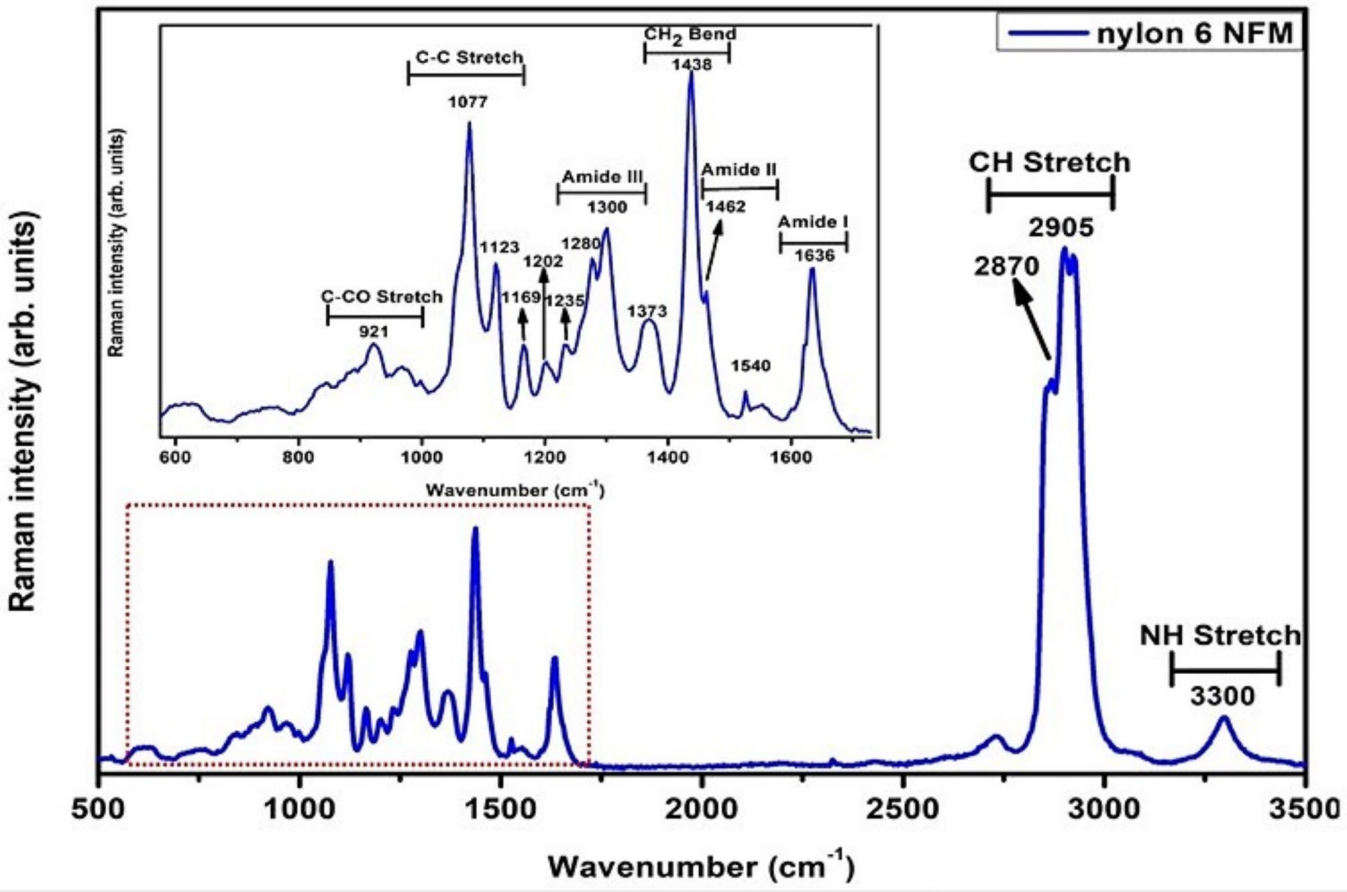
Raman spectrum of electrospun Nylon 6 nanofiber.

For higher spatial resolution, confocal Raman spectroscopy investigation was carried out to investigate the presence of overlapping peaks and vibrational modes in the material which are present but perhaps obscure using FTIR spectroscopy. The Raman spectrum in Fig. 6 was obtained at room temperature using a WiTec alpha 300RA + confocal Raman microscope and a laser wavelength of 532 nm as the excitation source. Some of the vibrational modes which were detected in the present study using Raman spectroscopy but were obscure in FTIR include the C-C stretching vibrations at 1077 cm^− 1^ and 1123 cm^[− 1 41, 39^. Other peaks which were conspicuous in the Raman spectrum are the C-N stretching and the N-H bend also known as the amide III region at 1280 cm^− 1^ and 1300 cm^[− 1 41, 39^. A strong band which is attributed to the CH_2_ bend, and suggestive of the trans amide group was observed at 1438 cm^− 1 41^. The broad band observed at 1373 cm^− 1^ is the wagging mode^40,41^ originating from the CH_2_ wag vibrations, while the amide I mode was observed at 1636 cm^− 1^, and primarily attributed to the C = O stretching^39^. The amide II Raman transition, a combination of C-N stretch and N-H bend is seen as a shoulder at 1462 cm^− 1^ in the spectrum. The weak frequency at 1540 cm^− 1^ (more visible in the FTIR spectrum) can be described as part of the amide II transition due to the N-H stretching and C = O in-plane bending vibrational modes^38^. The band at 1169 cm^− 1^ is assigned to be as a result of the CH_2_ twist vibrations^41^, while the frequencies at 1202 cm^− 1^ and 1235 cm^− 1^ are a combination of the CH_2_ wag and the CH_2_ twist vibrations^41^. A prominent peak corresponding to the C-CO stretching vibrations was also observed at 921 cm^− 1 42^, while the bands at 3300 cm^− 1^ and 2870–2905 cm^− 1^ are from the N-H stretching and C-H stretching respectively.

### Theoretical studies of physicochemical properties of Nylon 6 NFM

**Fig. 7:**
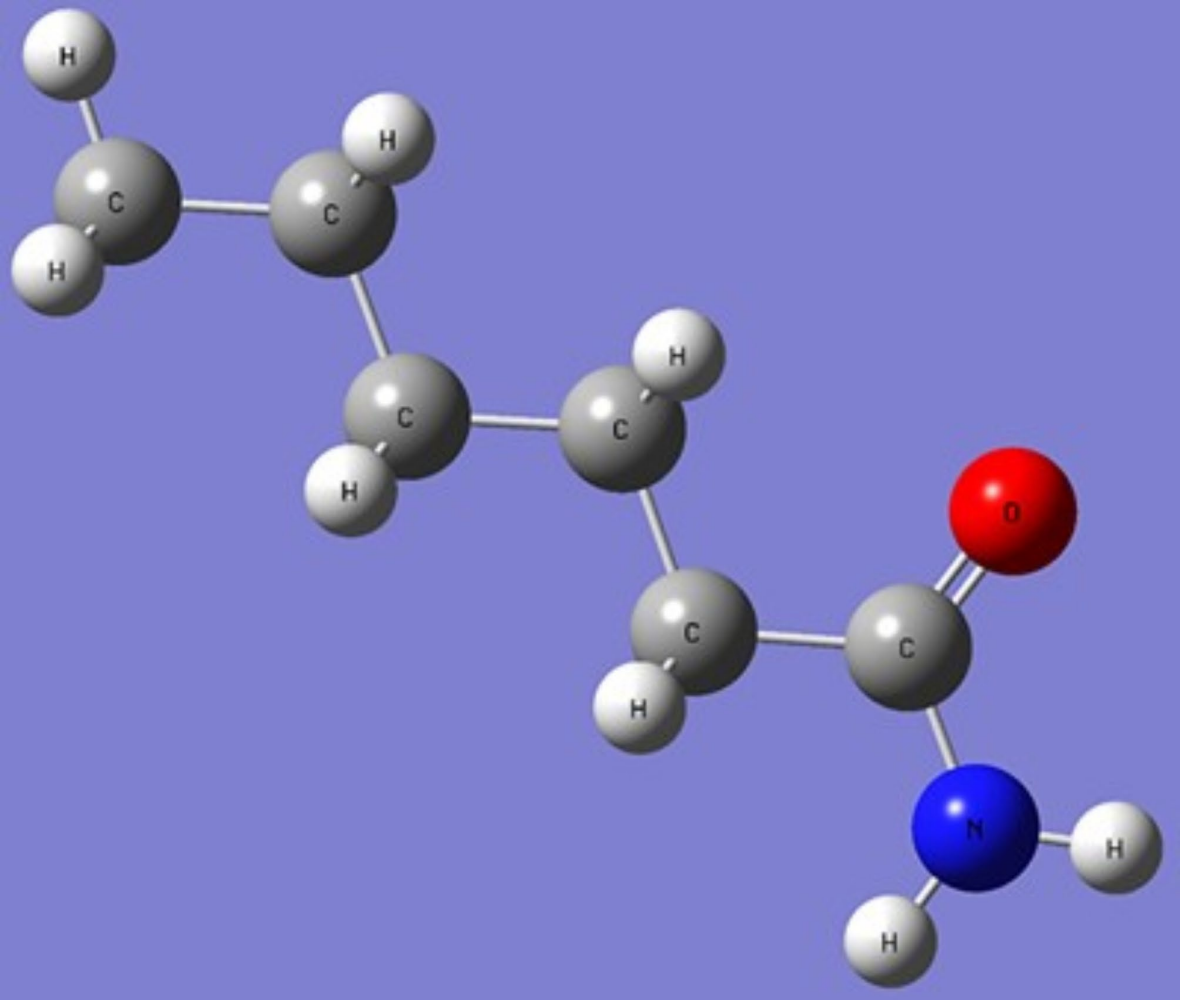
Optimised molecular structure of Nylon 6 monomer.

Figure 7 displays the optimised configurations for the investigated nylon 6. The quantum chemical data utilised to elucidate the physicochemical characteristics and compare with experimental outcomes are exclusively related to the compound’s lowest-energy conformer being investigated. According to the frontier molecular orbital theory, the interactions between the HOMO and LUMO of the interacting species have a substantial impact on chemical reactivity^42^. In addition, several quantum chemical parameters were computed to obtain a more profound understanding of the intermolecular interactions and optoelectronic features relating to nylon 6 nanofiber membranes (NFM).

### UV-VIS absorption spectroscopy

**Fig. 8:**
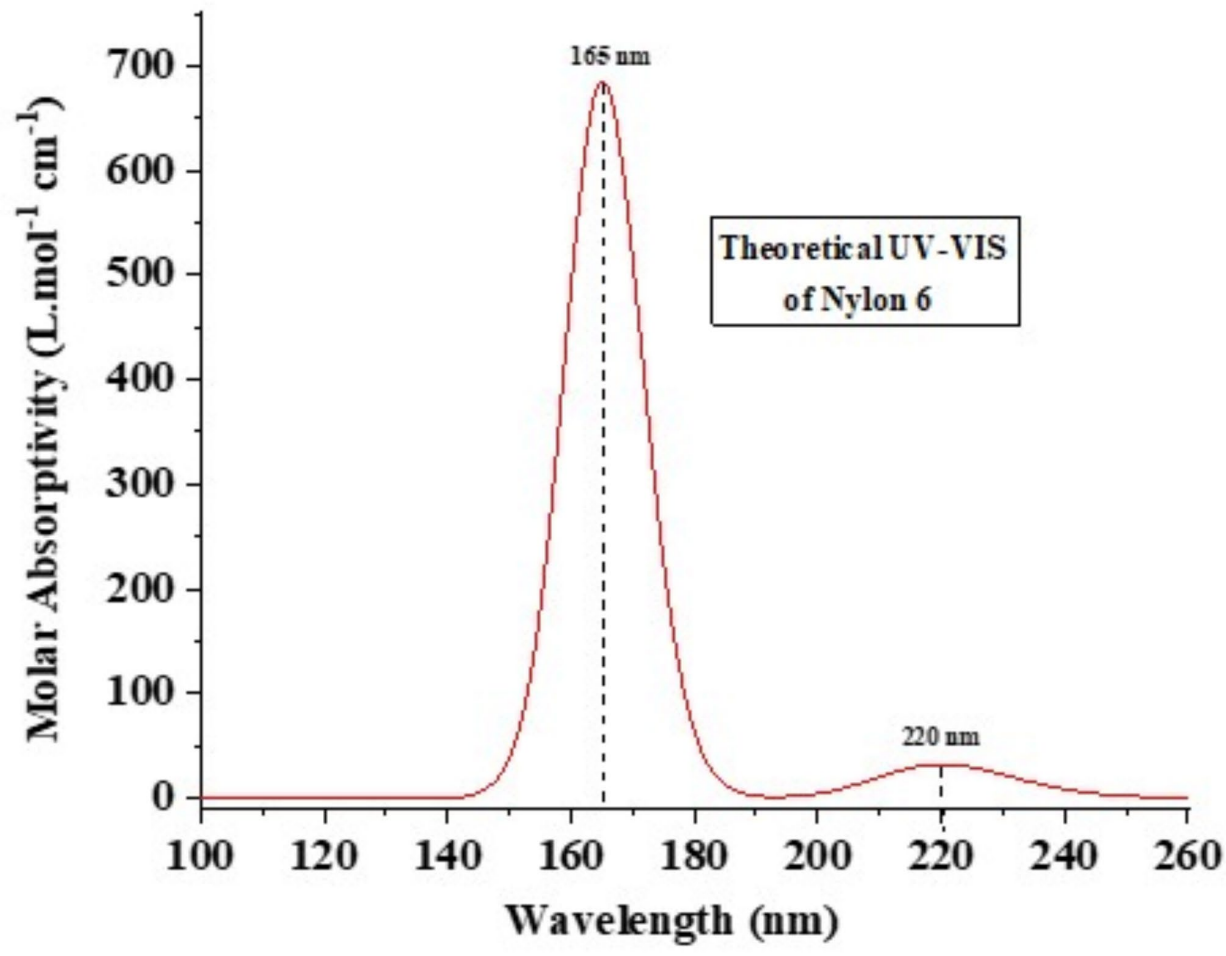
Theoretical UV-vis spectra of Nylon 6 monomers.

Theoretical modelling of the optoelectronic properties was performed to determine the critical absorption bands of the studied nylon 6 NFM. The analysis employed the TD-DFT/B3LYP approach using a 6-311 + + G (d, p) basis set under vacuum conditions (Fig. 8). Given the difficulties in directly measuring the UV-visible spectrum of nylon 6 NFMs, employing this theoretical method is the most effective way to study the optical characteristics of these polymers. The simulation forecasted two prominent electronic absorption peaks: the initial one being a substantial singlet excitation occurring at a wavelength of 165 nm, and the second one indicating electronic transitions between molecules, occurring at a wavelength of 220 nm. These results emphasise the individual contribution of each orbital to the observed spectrum.

### Frontier molecular orbital analysis

The molecular orbital energies, specifically HOMO and LUMO, offer crucial insights into the chemical species’ reactivity. The E_HOMO_ is commonly linked to the molecule’s electron-donating capacity^43^. A higher E_HOMO_ value signifies a greater inclination of the compound to transfer electrons to a suitable recipient species possessing minimal energy and unoccupied or partly occupied atomic or molecular orbitals. Table 4 represents the data that indicates that NFM exhibits a significantly elevated E_HOMO_ value, suggesting a strong inclination of NFM to donate electrons. The strong electron-donating behaviour of the compound can be attributed to the presence of substituents containing -NH groups, which can transfer electronic charge to the system. However, the -C = O group has electron-withdrawing properties. Consequently, the molecule of nylon 6 exhibits a dipole.

**Fig. 9:**
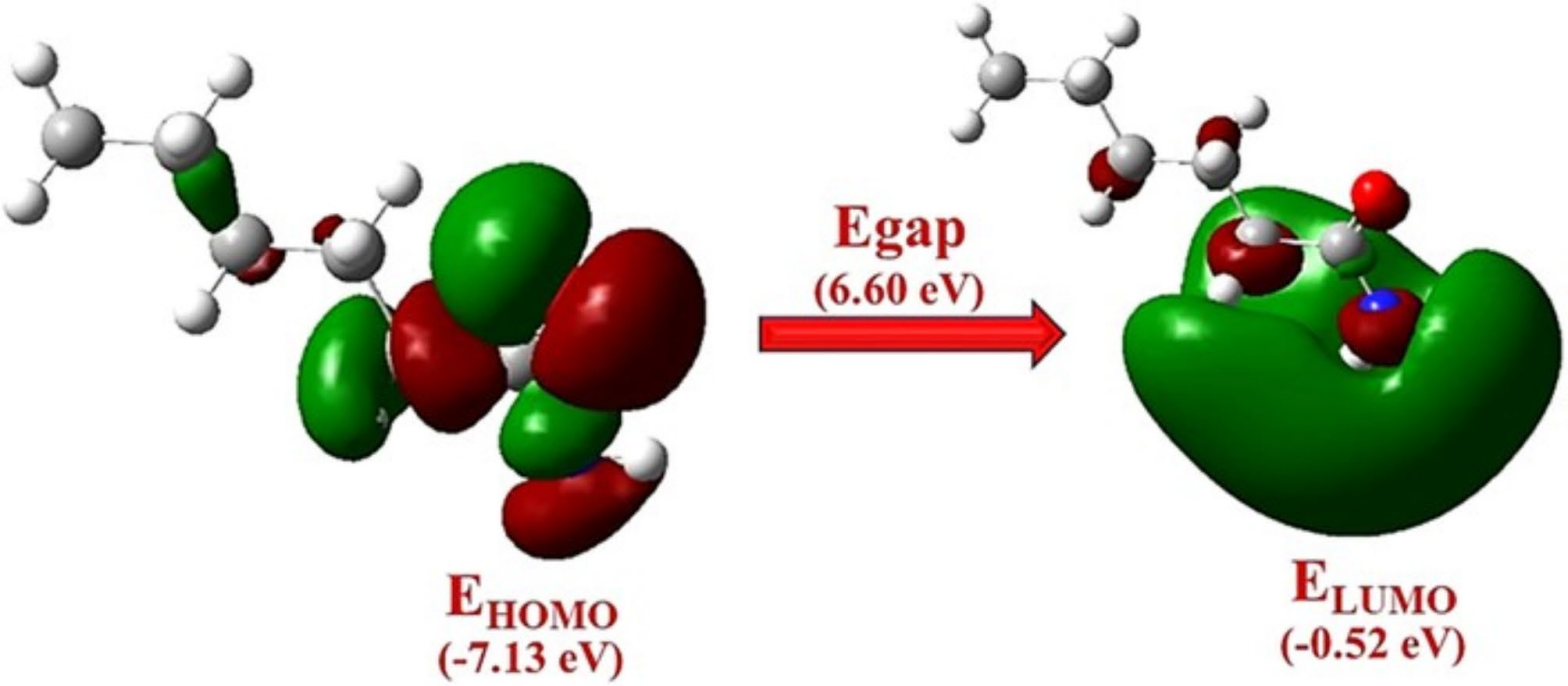
Analysis of the molecular orbitals and band gap energy (Egap) of Nylon 6 molecules.

Moreover, the progression of an electron from the HOMO to the LUMO indicates the absorption of electronic energy that occurs when transitioning from the lowest energy state to the first higher energy level. The high levels of first and second hyperpolarizability, which quantify the nonlinear optical (NLO) activity of a molecular system, are linked to the shift of electron clouds from donor to acceptor groups by intermolecular charge transfer. The HOMO is fundamentally localised in the vicinity of the amide group, while the LUMO is mainly localised in the vicinity of the amine-methylene moiety. This suggests that the transfer of charge occurs from the amide group to the amine-methylene moiety via the hydrogen bond, which is a crucial condition for achieving a significant second-order nonlinear optical (NLO) response. The optical gap is represented by the difference between the HOMO energy level at -7.13 eV and the LUMO energy level at -0.52 eV, which translates to a gap of 6.60 eV. The electronic arrangement of the HOMO and the LUMO energy levels is seen in Fig. 9.

The observed reaction of nylon 6 NFM indicates that it has the potential to be used as a material for sensing and absorption. This is because it can easily release electrons towards a modified electrode when a potential is applied, while an analyte existing in the solution can take these electrons during reduction. The argument is further supported by the electronegativity values (X) of the compounds, as indicated in Table 4. Furthermore, the HOMO-LUMO energy gap (ΔE) for nylon 6 NFM is determined to be 6.60 eV, suggesting a higher likelihood of the adsorption of the analyte molecule^44^.

The global reactivity descriptors are employed for the prediction of global reactivity trends. The HOMO-LUMO energies were used to compute several chemical reactivity characteristics. The HOMO corresponds to the exterior orbital responsible for holding electronic charges and is strongly linked to the ionisation potential. Conversely, the LUMO is the core orbital that lacks any electrons and is related to electron affinity. By applying Koopmans’ theorem and Eqs. 1–4, we computed several parameters including chemical hardness, electron affinity, electronic chemical potential, chemical softness, global electrophilicity index, and ionisation potential which are commonly represented as (ɳ, A, µ, S, ω, and I) respectively^21,45^. The frontier molecular energy gap is essential in assessing the chemical reactivity and stability of a molecule.

The calculated quantum chemical analysis was correlated with experimental studies of nylon 6 to enhance understanding of the material’s electronic behaviour and reactivity. The elevated calculated energy gap of 6.60 eV indicates that nylon 6 has the potential to effectively participate in a redox reaction. The study by Maleki et al.^46^ supports this prediction, concluding that nylon 6 demonstrates significant electrochemical behaviour, specifically its redox properties as indicated by the energy gap, thereby placing it as a potential candidate for sensor applications. They have demonstrated significant responsiveness in the detection of various analytes. The global reactivity descriptors obtained from HOMO-LUMO energies reinforce the correlation between theoretical predictions and experimental results^47^. The correlation between chemical hardness and softness values indicates favourable kinetic stability, aligning with calculated chemical hardness values and reflecting a balance between reactivity and stability. A tendency towards electrophilic interactions, as indicated by the electrophilicity index obtained from DFT calculations, has been noted experimentally in reactions involving nylon 6 derivatives^46^. In comparison to the previously published work^48^, the computed findings presented in Table 4 demonstrate that the chemical exhibits favourable kinetic stability.

1 $$\:\eta=\frac{1}{2}\left(\text{I}\:\--\text{A}\right)$$

2 $$\:S=\frac{1}{\eta\:}$$

3 $$\:{\upmu\:}\:=-\frac{1}{2}\left(\text{I}+\text{A}\right)$$

4 $$\:{\upomega\:}=\frac{{{\upmu\:}}^{2}}{2{\upeta\:}}\:$$

**Table 4:** HOMO-LUMO energies, reactivity properties and corresponding estimated values for Nylon 6-molecule.

Molecular orbital energies and Quantum chemical properties (eV)	Estimated Values
E _LUMO_	-0.52
E _HOMO_	-7.13
ΔE = E_LUMO_-E_HOMO_	6.60
Global electrophilicity Index (***ω)***	2.22
Electronic chemical potential (***µ)***	-3.83
Chemical Softness (***S)***	0.30
Global Hardness (***η)***	3.31
Ionization Energy **(*I)***	7.13
Electron affinity (***A)***	0.52

### Molecular electrostatic surface potential (MESP)

**Fig. 10:**
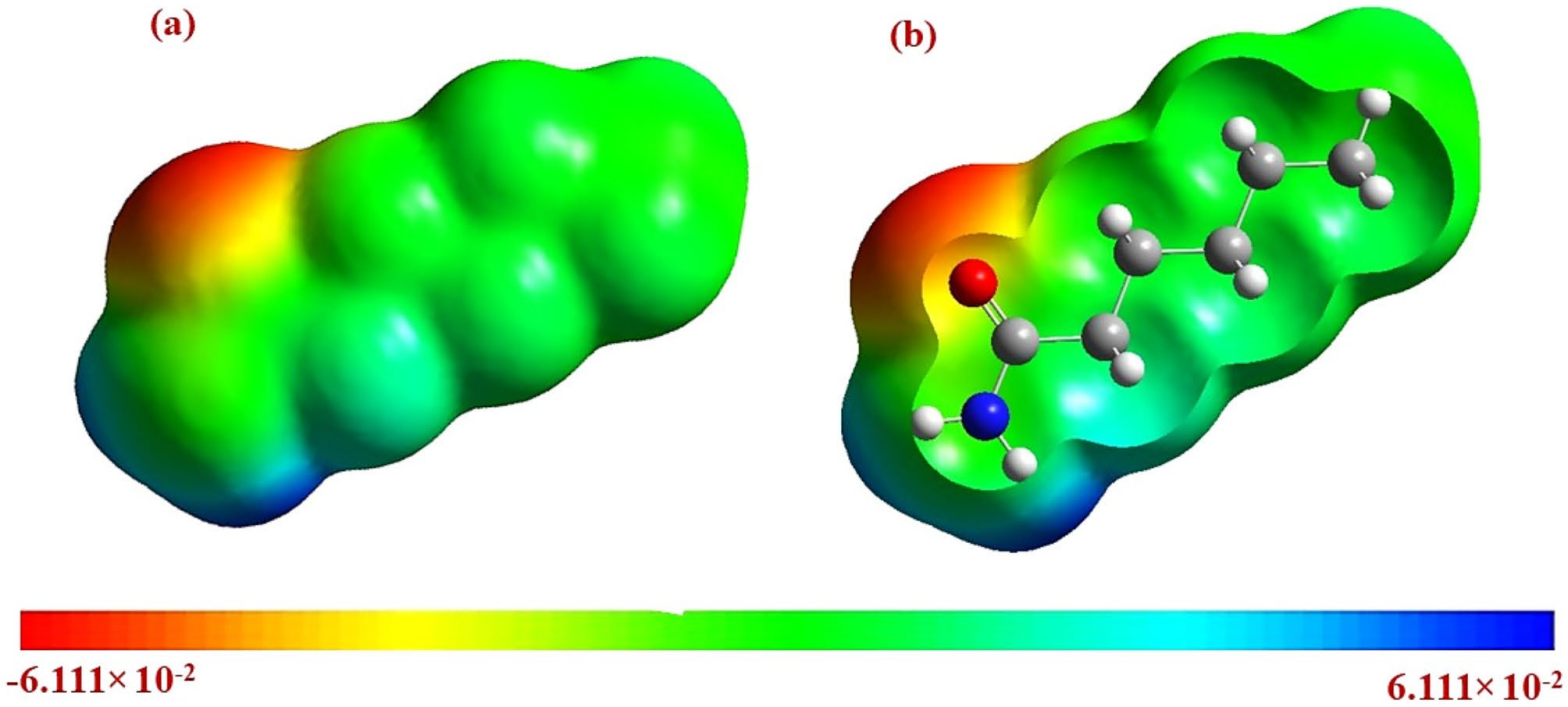
The MESP mapping of Nylon 6 molecule: (**a**) zipped MESP (**b**) unzipped MESP.

Figure 10 illustrates the MESP for nylon 6 NFM. The electrostatic potential provides an efficient way to illustrate the characteristics and behaviour of atoms and molecules in terms of their charge distribution^49^. The data is graphed on a surface with a consistent electron density. The MESP was used to establish the chemical reactivity of sites and the polar character of Nylon 6, as evidenced by the value 3.6259 Debye, indicating its dipole moment. The MESP calculation was employed to forecast the occurrence of sites of reactivity in the Nylon 6 compound, indicating potential vulnerability to electrophilic and nucleophilic attack. The various colours on the MESP graphic correspond to distinct electrostatic potential levels. The sections coloured in red, blue, yellow and green correspond to the highest levels of electrophilic reactivity, nucleophilic reactivity, and zero electrostatic potential, respectively. The potential decreases in the corresponding order: blue > green > yellow > orange > red. Concerning nylon 6 NFM, the positive charge is mainly distributed around the nitrogen atom of the amine group, which is a favourable location for electrophilic attack. The negative charge is primarily distributed over the atomic oxygen of the carbonyl (C = O) group in the polymer, making it susceptible to electrophilic assault. The observation indicates that the charge delocalization in the nylon 6 molecule is regulated by an association between the regions of the molecule that have an excess of electrons and those that are electron deficient. parts of the molecule. This contact facilitates the passage of carriers on the polymer. Considering the framework of the nylon 6 structure, the negative electrostatic potential enhances the carrier transport behaviour of nylon 6 NFM^50^. According to the visual MESP plot, it can be confidently said that nylon 6 NFM has a strong ability to transfer charges when used in electrochemical sensing or electrical devices.

## Conclusion

The present study demonstrates the successful fabrication of Nylon-6 nanofibers by the process of electrospinning. We employed the DFT technique, (B3LYP) with a basis set of 6-311 + + G(d, p) to perform molecular optimisation and calculate chemical descriptors, including HOMO-LUMO, ionisation energy, electron affinity, etc. for the molecule in its ground state. The nylon 6 solution was prepared with three different concentrations: 14 wt%, 16 wt%, and 18 wt%. The solution was subjected to different voltages, specifically 20 kV and 25 kV. The study found that the inclusion of 14 wt% nanofibers promote the development of consistent ultrafine nanofibers, eliminating the presence of beads and spider-net structures. The diameter of the electrospun fibres is directly proportional to the solution concentration and exhibits a little reduction with higher voltage. As the applied voltage reached 25 kV, the fibre diameters exhibited a reduction as the concentrations increased. The thickness of the electrospun membranes diminishes as the electrospinning voltage increases, making it challenging to create such membranes. The amine group’s nitrogen atom was identified as the binding site for electrophilic assault by the examination of the MESP. The Nylon 6 ‘s band energy difference between the HOMO, LUMO, and global chemical reactivity characteristics indicate its kinetic stability. The findings of this study demonstrate that the variations in concentration and applied voltage are crucial factors in altering the characteristics of electrospun nanofibers.

## Data Availability

All data are in the manuscript.
